# Benefits of a Skull‐Interfaced Flexible and Implantable Multilight Emitting Diode Array for Photobiomodulation in Ischemic Stroke

**DOI:** 10.1002/advs.202104629

**Published:** 2022-01-25

**Authors:** Hyunha Kim, Min Jae Kim, Young Woo Kwon, Sangheon Jeon, Seo‐Yeon Lee, Chang‐Seok Kim, Byung Tae Choi, Yong‐Il Shin, Suck Won Hong, Hwa Kyoung Shin

**Affiliations:** ^1^ Department of Korean Medical Science Graduate Training Program of Korean Medical Therapeutics for Healthy‐Aging School of Korean Medicine Pusan National University Yangsan 50612 Republic of Korea; ^2^ Department of Pharmacology and Neuroscience Creighton University School of Medicine Omaha NE 68178 USA; ^3^ Department of Nano‐Fusion Technology College of Nanoscience & Nanotechnology Pusan National University Busan 46241 Republic of Korea; ^4^ Department of Congo‐Mechatronics Engineering Department of Optics and Mechatronics Engineering College of Nanoscience & Nanotechnology Pusan National University Busan 46241 Republic of Korea; ^5^ Department of Pharmacology Wonkwang University School of Medicine Iksan 54538 Republic of Korea; ^6^ Department of Rehabilitation Medicine School of Medicine Pusan National University Yangsan 50612 Republic of Korea

**Keywords:** light‐emitting diodes, photobiomodulation, poststroke cognitive impairments, stroke

## Abstract

Photobiomodulation (PBM) has received attention due to its potential for improving tissue function and enhancing regeneration in stroke. A lightweight, compact, and simple system of miniaturized electronic devices consisting of packaged light‐emitting diodes (LEDs) that incorporates a flexible substrate for in vivo brain PBM in a mouse model is developed. Using this device platform, the preventive and therapeutic effects of PBM affixed to the exposed skull of mice in the photothrombosis and middle cerebral artery occlusion stroke model are evaluated. Among the wavelength range of 630, 850, and 940 nm LED array, the PBM with 630‐nm LED array is proved to be the most effective for reducing the infarction volume and neurological impairment after ischemic stroke. Moreover, the PBM with 630 nm LED array remarkably improves the capability of spatial learning and memory in the chronic poststroke phase, attenuates AIM2 inflammasome activation and inflammasome‐mediated pyroptosis, and modulates microglial polarization in the hippocampus and cortex 7 days following ischemic stroke. Thus, PBM may prevent tissue and functional damage in acute ischemic injury, thereby attenuating the development of cognitive impairment after stroke.

## Graphical abstract

Photobiomodulation (PBM) shows promising results regarding the improvement of tissue function and poststroke regeneration. A miniaturized brain PBM system using an implanted multilight‐emitting diodes array with a wavelength of 630 nm results in tissue damage alleviation and infarction volume reduction; brain PBM therapy in the acute phase of stroke may contribute to recovering cognitive impairment after ischemic stroke.

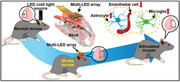

## 1 Introduction

Interest in tissue‐electronic interfaces has increased recently due to a variety of potential applications including light‐based therapy, electrophysiological tracking diagnosis, electrical stimulation, and the therapeutic implementations of optoelectronic devices.^[^ ^1^, ^3^ ^]^ Enabled by the conformal fixation of implantable devices to internal tissues,^[^ ^4^, ^5^ ^]^ on‐demand integration of advanced therapeutic devices with biological systems has accelerated basic scientific research and the respective translation into clinical medicine.^[^ ^5^ ^]^ The sophisticated designs, guided by biological analysis of the model systems, allow appropriate platforms to be placed onto soft and complex tissue surfaces of interest.^[^ ^7^, ^8^, ^9^ ^]^ Among other candidate platforms, optoelectronic devices for the local irradiation of sufficient light energy to target lesions comprise such systems, offering an effective approach.^[^ ^9^ ^]^ For example, photobiomodulation (PBM), also known as low‐level light therapy (LLLT), has received tremendous attention for the treatment of traumatic brain injury (TBI), stroke, wound healing, and spinal cord injuries, as well as for mitigating cancer therapy side effects, as it improves tissue function or enhances regeneration.^[^ ^11^, ^12^, ^13^ ^]^ In particular, PBM has been reported as an effective treatment for acute stroke, with potentially beneficial effects on the recovery of brain function. It has also been proven that transcranial PBM therapy within 24 h of stroke onset is safe for patients in terms of occurrence of adverse effects and mortality.^[^ ^14^, ^15^ ^]^ Therefore, it has been widely explored in preclinical studies evaluating the biological effects of PBM for the treatment of stroke using light sources with certain wavelengths and incident powers.^[^ ^15^ ^]^ Previous studies focusing on the neuroprotective effects of the PBM approach have relied on the evaluation of functional tests or mortality rates,^[^ ^14^, ^15^ ^]^ but the direct beneficial effects of PBM on recovery of a damaged neuronal tissue have not been thoroughly tested so far. To practically resolve internal lesions, light sources are applied into the brain interface—internally with an endoscope or externally with the aid of suitable materials—to provide intensive long‐term or short‐term photoirradiation. However, due to the limited control of the distance and area between the output ends of the light source, it is difficult to continuously illuminate the exact target position with the desired fluence rate during treatment.^[^ ^17^, ^18^ ^]^ Canonical PBM experiments, particularly in the brain, require cranial insertion of an optical component (e.g., optical fibers) to illuminate a region of interest.^[^ ^18^ ^]^

In this context, a new concept of microelectronic devices has been introduced; the conceptual integration safely interfaced with internal tissues can illuminate challenging target locations for advanced biomedical studies, which include not only the brain but also other organs involved in chronic pain or neurological disorders.^[^ ^20^, ^21^ ^]^ Representatively, miniaturized light‐emitting diode (LED) devices have emerged as an innovative light source for brain PBM for a wide range of neurological conditions.^[^ ^22^, ^23^ ^]^ The typical brain PBMs facilitated by LED devices involve exposure of neural tissue to a low fluence of light, ranging from ≈1 to 20 J cm^−2^, and at wavelengths ranging from red to near‐infrared (i.e., 600–1200 nm).^[^ ^23^ ^]^ Notably, the tissue penetration properties of red light (i.e., ≈600–880 nm), together with its high efficacy,^[^ ^23^ ^]^ have generally made it the most popular wavelength range, and some studies have demonstrated that LLLT applied to experimental animals after ischemia improved neurological rating scores via direct illumination through the scalp.^[^ ^23^ ^]^ Recent technologies based on microscale LEDs offer important functionality, and their benefits are now rapidly extending to wearable, implantable, and other biomedical applications.^[^ ^25^, ^26^ ^]^ Their expanded use in neuronal injuries has even been tested in pathologies such as TBI and stroke by employing the neuroprotectant approach in cell in vitro assays or animal in vivo studies. Observation of the therapeutic effects of PBM in various disease models is important since the same stimulation modality is preferred to translate the findings from preclinical to clinical trials. Therefore, a newly developed PBM system that can be easily adjusted for small animals—that is, a miniaturized brain PBM toolbox—may also facilitate investigation of the biological mechanisms of PBM, and promote its potential for continuous preclinical‐grade assessments in laboratory settings. As presented in our previous studies, the non‐invasive brain stimulation technique based on PBM could be useful for treating motor disorders, as well as for poststroke rehabilitation, through application to the scalp over a targeted cortical area.^[^ ^27^, ^28^, ^29^, ^30^ ^]^ However, previous experiments in small‐animal models, such as mouse models, have been limited in terms of stimulation, due to the inherent limitations of their designs and the size‐limitation of the probes.

Here, we demonstrate an implantable PBM system based on multi‐LED arrays that are stably in contact with the skull over a targeted cortical area, which enabled the study of the long‐term therapeutic effects. Taking the key advantages of the existing technology into account, we performed an in vivo mouse experiment to evaluate the efficacy of the LLLT for minimizing acute ischemic brain damage and chronic cognitive impairment after focal cerebral ischemia. The long‐term therapeutic effects of PBM on various disease models could be evaluated with suitable device fixation and the precise shining of light on brain lesions due to the advantageous tissue‐adhesive optoelectronic devices implemented on the flexible printed circuit board (FPCB) with optimal wavelength. Such capabilities would enable examination of biological mechanisms in model animals, a major goal of modern neurocognitive behavior research. The major contribution of brain PBM therapy may be associated with various biological processes, such as increasing the metabolic capacity of neurons and stimulating anti‐inflammatory, anti‐apoptotic, and antioxidant responses, as well as with neurogenesis and synaptogenesis.^[^ ^31^, ^32^ ^]^ Neuroinflammation is one of the crucial pathophysiological features of ischemic stroke and aging, which likely have a detrimental effect on cognitive function after stroke.^[^ ^33^, ^34^, ^35^ ^]^ In recent studies, we found that absent in melanoma 2 (AIM2) inflammasome‐mediated inflammation and pyroptosis contributed to chronic poststroke cognitive impairment (PSCI), and we reported beneficial pre‐conditioning effects of brain PBM therapy for the reduction of cortical tumor necrosing factor‐*α* and interleukin (IL)‐1*β* expression at 24 h postischemia.^[^ ^30^, ^36^ ^]^ Furthermore, brain PBM in a mouse photothrombotic stroke model reduced IL‐1*β* and IL‐18 levels at 72 h post stroke via inhibition of the NLRP3 inflammasome signaling pathway.^[^ ^26^ ^]^ Therefore, it is expected that brain PBM using an implantable optoelectronic device could attenuate the development of PSCI by controlling the inflammatory response. In addition, we first examined the preventive and therapeutic effects of brain PBM on infarct volume and performed behavioral assessments of neurological and motor functions after ischemic brain injury. Further, we also evaluated whether brain PBM therapy in the acute phase of stroke attenuates the development of PSCI by controlling AIM2 inflammasome activation and the inflammatory response. The aim of this study was to demonstrate the robustness of this miniaturized optoelectronic device implemented via brain implants and its practical utility for PBM therapy in live animals.

## 2 Results

### 2.1 Concept of an Implantable Multi‐LED Array for Mouse Model Studies

**Figure 1:**
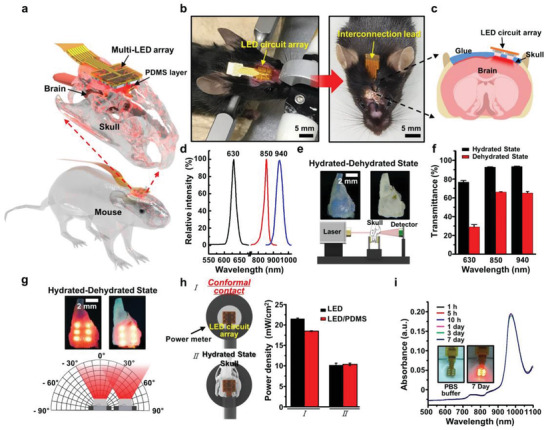
a) Schematic illustration of the skull‐interfaced multi‐LED array. b) The experimental design of the implantable multi‐LED device, conformally covering a local brain area for a mouse model study. The right image shows a fully implanted multi‐LED device except for interconnection leads. c) Schematic illustration of the LED circuit array placed outside the skull, above the brain. d) The relative intensity of three different wavelengths (i.e., 630, 850, and 940 nm), generated from the multi‐LED arrays used in the in vivo experiments. e) Digital images of mice skulls for the measurement of the transcranial transmission to determine the extent of LED light penetration across the skull barrier, and the measurement laser setup. f) The transmittance data measured from hydrated/dehydrated skull samples. g) Digital images of the illuminating six‐LED cores of the device, and the angle‐dependent spatial distribution of light sources. h) The measured surface power density of multi‐LED array with or without PDMS encapsulant. i) The reliability test of PDMS encapsulant for the multi‐LED array.

**Figure** **1**a presents a schematic illustration of the skull‐interfaced multi‐LED array integrated on a flexible substrate (i.e., polyimide, PI), highlighting an extremely simple concept for replaceable prostheses. The photographs in Figure 1b capture the experimental design of the implantable optoelectronic system that can cover a local contact area on the brain for mouse model studies. The fully flexible and implantable system enables accurate local irradiation with the light source within a restricted range and an easy‐to‐plug configuration that provides diffusive light channels through the skull as schematically described in Figure 1c. To secure the flexible multi‐LED array on the skull, a thin layer of biomedical glue (≈1.3 µm) was used to prevent unwanted detachment in repeated attempts by the mouse to remove it with its paws upon sensing a foreign body.^[^ ^36^ ^]^ In our PBM system, three different wavelengths were carefully selected and generated from the LED circuit, including 630, 850, and 940 nm, to evaluate the potential efficacy of the therapeutic approach (Figure 1d).^[^ ^37^ ^]^ As the improvement effects of the PBM may be dependent on the quantity of LED light that crosses the skull barrier, transcranial transmission was investigated to determine the extent of LED light penetration across the skull barrier using the laser setup and photodetector diagrammatically represented in Figure 1e.^[^ ^25^ ^]^ In this experiment, skulls were dissected, and comparisons were performed between hydrated and dehydrated skulls (thickness ≈ 0.26–0.27 mm) to evaluate light penetration in an ambient condition with a power density of ≈17 mW cm^−2^ (Table S1, Supporting Information). At all wavelengths, the hydrated state allowed for higher penetration of laser radiation due to the existence of water. A statistically significant decrease in penetration (≈41%) was detected for the wavelength of 630 nm, as shown in Figure 1f. When the multi‐LED array was tested with conformal contact with the skull, the apparent penetration profiles differed between the hydrated and dehydrated states, as shown in Figure 1g. Extensive diffusion and scattering patterns were observed in‐and‐around the centers of the illuminated six‐LED cores.

As the light passes through a heterogeneous and opaque environment such as living tissues, the trajectory of the incident light is dynamically deviated by the barrier component. Thus, only a partial fraction of light waves propagating inside biological tissues can reach the desired target area, while most are scattered and randomly diffused.^[^ ^38^ ^]^ In this regard, the hydrated skull serves to diffuse the light, attenuating it, but effectively covering the light (i.e., light diffuser) over a target area. Different diffused areas are shown in the photograph (top panel in Figure 1g). Moreover, the spatial directivity of LED radiation intensity can be estimated by applying a multi‐LED directivity pattern in wide‐angle responses defined by the average from adjacent individual LEDs, as schematically represented in the bottom panel in Figure 1g.^[^ ^39^ ^]^ The surface power density was measured by a power meter (PM100D; Thorlabs, Newton, NJ, USA) with a skull‐mounted multi‐LED array, as featured in Figure 1h. In a view of the real application, this measurement can be a practical guide for the light‐dose per treatment area. The gradual attenuation of light penetration was naturally expected because of the hydrated skull barrier and an encapsulant (i.e., transparent elastomer, polydimethylsiloxane, PDMS: film thickness = 700 µm) wrapping the electric circuit.^[^ ^40^ ^]^ Thus, the density of power delivered to the cortical surface would be decreased with respect to the series of compartmentalized barriers prior to producing a biological effect. Additional barriers such as the encapsulant (i.e., PDMS) and the skull affected the overall density of the performance of the multi‐LED array in the far‐field range (Figure 1h). The originally manufactured multi‐LED array exhibited a surface power density of ≈22 mW cm^−2^ when conformally contacted with the power meter, and appeared separately as ≈17 mW cm^−2^ in the encapsulated condition (Figure S1, Supporting Information). Although the PDMS packaging process decreased overall LED efficiency by ≈22.7%, encapsulation was inevitable for implantation. On the other hand, the hydrated skull barrier distance ranged ≈0.26–0.27 mm and resulted in a power density of ≈10 mW cm^−2^, which has been reported as a beneficial range of power density in thin‐skull‐based PBM animal studies (i.e., ≈10–25 mW cm^−2^), leading to an optimized trial design in our experimental approach. In addition, to test the degree of the chemical and water‐proof properties of the multi‐LED array in the biological environment (i.e., hydrated condition), the device was placed in phosphate‐buffered saline (PBS) buffer solution (pH = 7.4) at 37 °C for 7 days as demonstrated in Figure 1i.^[^ ^3^ ^]^ The absorbance was continuously measured using ultraviolet‐visible spectroscopy to detect possible dissociated molecules from the encapsulated LED device affected by the changes in the pH of the PBS buffer solution. PDMS encapsulation exhibited superior chemical stability; no changes were found, as shown in the graph. Clearly, the inset photograph of the luminous multi‐LED array soaking in the PBS buffer solution is presented in the inset after 7 days, delivering the same power density of ≈10 mW cm^−2^, without degradation in its performance.

### 2.2 Materials and Design of the Multi‐LED array for a Mini‐PBM Module

**Figure 2:**
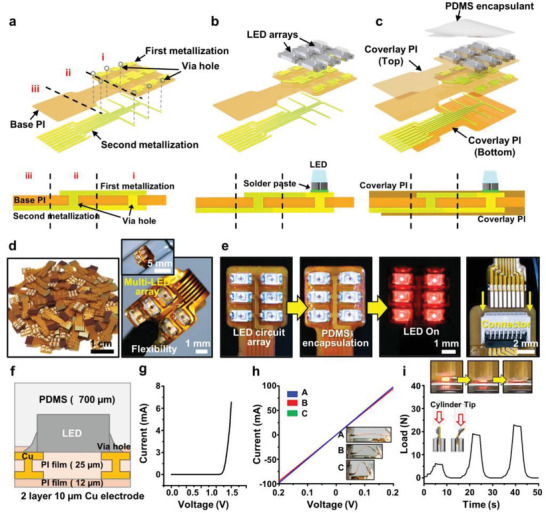
a–c) Schematic illustration of the fabrication of the multi‐LED array, built with a close‐packed electrode design on a flexible PI substrate including interlayer and coverlay PI, and a double‐stacked Cu electrode. d) Digital images of the monolithically integrated LED chips, demonstrating deformability in specific conditions. e) Operated multi‐LED array, an encapsulated state with PDMS. The right panel shows the plugged end of leads with a connector. f) Side view of an LED chip mounted on the holes. g) Operating current/voltage of a single LED chip. h) Stability test of the connected multi‐LED arrays with external leads. i) Load resistance test in pressure and bending of the multi‐LED array.

**Figure** **2**a–c shows schematic illustrations of the layout and fabrication procedure of a mini‐PBM module (see more information in Figure S2 in the Supporting Information). Main LED components on the flexible substrate were assembled by surface‐mount technology (SMT), which provided easy access to realize our concept without the limit in designing an electrically and mechanically reliable PBM module.^[^ ^42^, ^43^ ^]^ The first basic embodiment adopts through‐hole via based electric interconnections in the shape of a vertically penetrating configuration, built in a thin and flexible substrate of 25‐µm‐thick PI. The relatively wide via‐hole geometry of the FPCB provides simultaneous electrical input channels through sequential first and second metallization to an array of the LED components that can only be contacted on the top electrodes to avoid a complex interconnection design, as shown in Figure 2a. This is the main scheme in the architecture of the integrated LED circuit, which can enable miniaturization of the PBM module by separating the crowded passive electrical paths to the opposite side in a single layer circuit board.^[^ ^43^ ^]^ Compared with the designs of other optical units assembled in a planar configuration, the slightly changed form factor could reduce the actual size of the PBM module; this allows for some marginal bending, precisely fitting it on the target size of the mouse brain. At the initial stage of the process, with the double‐sided thin metal films (1‐µm‐thick Cu) provided on the PI board, conventional photolithography was used to separately define the top contact pads for LEDs and the lower electrical interconnects by a precise mask‐alignment. Next, individual micro‐packaged LEDs (Wurth Electronics, Niedernhall, Germany) were manually placed on the contact pads joined with the solder paste (lead‐free solder alloy) as described in Figure 2b. Subsequently, a reflow process was performed in an oven at eutectic temperature (≈250 °C) to induce the self‐alignment by complemental wetting and to create permanent solder joints. After the SMT process of the LED components, a thin overlay (12 µm, PI) conformed and bonded to the top and bottom plane in a multilayer format to protect the exposed surface of the electrodes on the circuit. Moreover, this lay‐up process could provide some additional physical capabilities, such as electrical insulation, bending resistance, and heat spreading, including the length tailoring in the exposed electrical ends to the connector (Figure 2c). Next, a water‐proof encapsulant of transparent PDMS elastomer (modulus of ≈100 kPa) was applied on the upper side of the surface‐mount LEDs to allow for a chemically stable operation in a biological environment by preventing possible dissociation from the solder joints.^[^ ^42^ ^]^ This final step also has the extra function of filling the bumps and flattening the uneven corrugated LED surface. When contacting the LED‐side on the mouse skull, the surface flatness can be one of the key elements for conformity and favorable tackiness. Since previous studies of the constituent materials (PI and PDMS) have revealed no evidence of toxicity, chemical decoupling from the provided support‐base would represent chronic stability to study PBM in the implanted area using the prepared optical stimulation array. In a well‐established manufacturing process with clear functionality, the lab‐scale mass production of mini‐PBM modules is suitable for a variety of mouse model studies and other related research in an implantable device format, as demonstrated in Figure 2d (≈100 pieces of modules). The mechanical durability of the multi‐LED circuit was simply tested by bending and folding in the physically harsh conditions, associated with the flexible format that well‐matched with the soft brain tissue (the right panel in Figure 2d). While the encapsulated LED illuminates a field of light centered on 6‐LED sources (Figure 2e), the maximum surface temperature rises slowly over a period of 10 min in the range of ≈34–35.8 °C during surgery (Figure S3, Supporting Information). This excellent thermal stability of the packaged multi‐LED array can be attributed to the effective intrinsically embedded heat sinks (i.e., interlayer and overlay of PI, double stacked Cu electrodes, Figure S4a, Supporting Information) and the heat dissipation through the top‐coated PDMS layer (Figure 2f, side view of the detailed structure of the monolithic LED component, a total thickness of ≈737 µm). The typical diode characteristic of the LED was measured under the repetitive bending cycle conditions to confirm its reliability in a flexible‐shaped configuration, as shown in Figure 2g. Current input and output voltages were evaluated under a bending and slight stretching state as presented in Figure 2h, where the connected leads (length = 300 mm) with a connector (Molex, Lisle, IL, USA) were firmly plugged, as shown in Figure 2e and Figure S4b–d (Supporting Information). Moreover, the extreme case was also tested revealing the load resistance as show in Figure 2i. As presented, owing to the mechanical compliance and advanced form factor, our multi‐LED array exhibited a significant reduction of the burden of processing with the viable combination of the soft materials and rigid electronic components.^[^ ^41^ ^]^

### 2.3 Brain PBM Assessment of Acute Brain Damage Following an Ischemic Stroke

**Figure 3:**
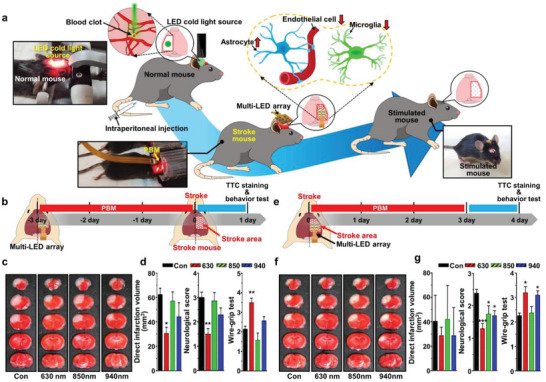
Experimental schematic diagram of the preventive and therapeutic effect of various wavelengths of brain PBM using multi‐LED array on the infarct volume and neurological and motor function after ischemic stroke. a) A representative photothrombotic stroke mouse model and the position of the multi‐LED array. b–e) Experimental procedures for brain PBM treatment and functional outcome tests after ischemic stroke. Mice receive three different wavelengths of 630, 850, or 940 nm (≈17 mW cm^−2^, 20 min), twice a day for 3 days, prior to or after the induction of ischemic insult. c,f) Representative photographs of coronal brain sections stained with TTC. d–g) Quantification of direct infarct volume and neurological score and wire‐grip results are evaluated to assess recovery of neurologic deficit and vestibular motor function after ischemic brain injury. All data are represented as mean ± SEM. *N* = 7–11 each. Statistical significance was determined by one‐way ANOVA with Tukey post hoc test. *p < 0.05, **p < 0.01, and ***p < 0.001 versus control group (Con).

The fabricated multi‐LED array was applied to a stroke mouse model for brain PBM therapy. We first examined the preventive effect of brain PBM using a multi‐LED array on the tissue and the functional outcomes following focal cerebral ischemia (**Figure** **3**). In this study, we adopted photothrombotic occlusion of cerebral microvessels in mice, which is based on a photochemical reaction triggered by systemic administration of Rose Bengal and focal illumination of the skull. Illumination leads to local activation of Rose Bengal, which results in free radical formation, disturbance of endothelial function, and local thrombotic occlusion of small cortical vessels (Figure 3a). The illuminated area occupied the sensorimotor cortex which indicated that the lesion would largely destroy the forelimb cortex and hence produce a deficit in the use of the forepaws. Mice underwent PBM therapy at different wavelengths of 630, 850, or 940 nm (≈17 mW cm^−2^, 20 min, twice a day for 3 days) prior to the induction of ischemia as well as 1 h prior to the procedure (Figure 3b). Thus, the outcome of the acute injury was evaluated on day 1 after focal cerebral ischemia. Notably, as shown in Figure 3c,d, triphenyl tetrazolium chloride (TTC) staining revealed that PBM therapy employing an LED array with a 630 nm wavelength significantly reduced direct infarct volume, compared to the control cases, which was measured to be 30.6 ± 5.0 and 62.4 ± 5.0 mm^3^ for PBM at wavelengths of 630 nm and control, respectively (*p* < 0.05 (*p* = 0.046)), whereas other PBM therapy with light sources of 850 and 940 nm had no significant effect. In addition, the mice treated with 630 nm PBM therapy showed significant improvement in neurologic and motor functions, suggesting that the reduced infarct volumes translated into better functional outcomes in the PBM‐treated group at wavelengths of 630 nm (Figure 3c,d).

Subsequently, we evaluated whether postischemic treatment of brain PBM with a multi‐LED array exerts a therapeutic effect on acute ischemic brain injury. The mice underwent PBM therapy (20 min, twice a day for 3 days), commencing at 4 h after the ischemic insult (Figure 3e). The PBM therapy at the wavelengths of 630 and 940 nm critically improved neurologic and motor function, compared to the control cases when measured 72 h after ischemic brain injury. However, the infarct volume was not reduced by the brain PBM, as presented in Figure 3f,g. The above combined results indicate that the ipsilateral cortical application of PBM, specifically with a wavelength of 630 nm, had not only a preventive effect on the tissue and the functional outcomes but also a therapeutic effect on the functional recovery after ischemic brain damage.

### 2.4 Brain PBM Assessment for Glial Cell Activation After Ischemic Brain Injury

**Figure 4:**
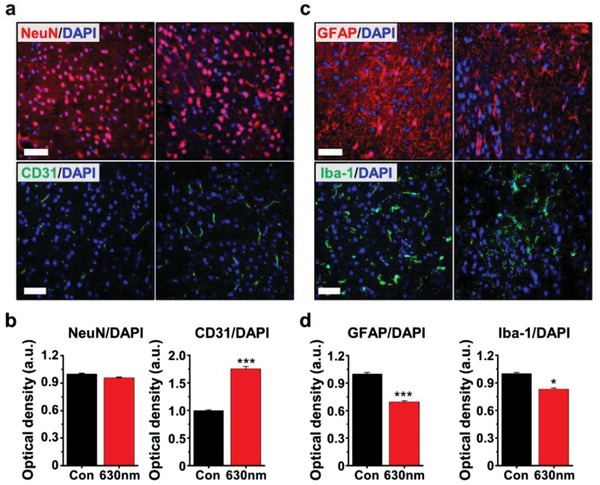
Effect of 630 nm brain PBM using multi‐LED array on brain cells following ischemic brain injury. Mice were pretreated with 630 nm PBM (≈17 mW cm^−2^, 20 min) twice a day for 3 days prior to the induction of ischemic insult. a,b) Representative photographs and quantification graphs of NeuN (red, neuronal marker) and CD31 (green, endothelial cell marker). c,d) Representative photographs and quantification graphs of GFAP (red, astrocyte marker) and Iba‐1 (green, microglia marker) at the peri‐infarct regions. DAPI is labeled with blue fluorescence. Scale bar = 50 µm. All data are represented as mean ± SEM. *N* = 4 each. Statistical significance was determined by unpaired, two‐tailed Student‘s *t*‐tests. *p < 0.05 and ***p < 0.001 versus control group (Con). Abbreviations: LED, light‐emitting diode; PBM, photobiomodulation; GFAP, astrocyte marker; DAPI, 4′,6‐Diamidino‐2‐phenylindole; NeuN, neuronal marker; CD31, endothelial cell marker; Iba‐1, microglia marker; SEM, standard error of the mean.

To evaluate the effectiveness of the PBM therapy, the brain cells in the ischemic cortex were examined by the measurement of the expression levels of the immunostained markers, such as NeuN (neuronal cell), CD31 (endothelial cell), GFAP (astrocyte), and Iba‐1 (microglia), at the peri‐infarct regions using fluorescence microscopy as presented in **Figure** **4**. The results indicate that neurons were not affected by the brain PBM at a wavelength of 630 nm, whereas the numbers of CD31^+^ cells were significantly increased in the PBM‐treated cases, compared to the control groups (Figure 4a,b), which implies that brain PBM treated with a 630 nm wavelength helped to preserve blood vessels in the ischemic area. Moreover, numerous astrocytes and microglia in the ischemic cortex were significantly decreased in the PBM‐treated groups (Figure 4c,d). To evaluate the level of effectiveness of PBM treatment, the same experiment was performed on the normal brain without any stroke. Interestingly, neurons and astrocytes were not affected by the brain PBM, but the numbers of CD31^+^ and Iba‐1^+^ cells were slightly affected by the brain PBM at the wavelength of 630 nm, as presented in Figure S5 in the Supporting Information. These results suggest that brain PBM decisively attenuates glial cell activation in the ischemic brain, whereas it increases blood vessels and decreases microglia activation even in the normal brain.

### 2.5 Brain PBM Assessment of PSCI Using a Multi‐LED Array

**Figure 5:**
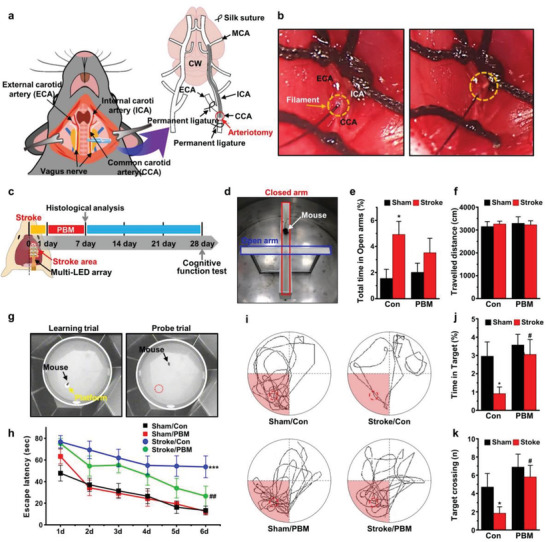
Effect of 630 nm brain PBM using multi‐LED array on cognitive dysfunction following ischemic brain injury. a) Cartoon of the MCAO/reperfusion mouse model. b) The microscopic appearance of a silicon‐coated 7‐0 monofilament insertion from the CCA to the ICA to occlude the MCA. c) Schematic diagram of experimental PBM procedure using a multi‐LED array in a PSCI model. Mice are treated with 630‐nm PBM (≈17 mW cm^−2^, 20 min) once a day for 7 days from next day after the ischemic insult. Histological analysis is performed at 7 days after MCAO and the cognitive functional tests are performed at 28 days after MCAO. d) Anxiety is measured using the elevated plus maze test. e) Total time spent in open arms and f) total traveled distance in the elevated plus maze for 10 min. g) Spatial learning and memory are assessed using Morris water maze test. On days 2–6, the mouse is placed in an opaque water‐filled pool (hidden platform) for learning trial tests, and on day 7, the mouse is allowed to swim freely without the platform for 90 s for the probe trial test. h) Average latency to find the visible platform on day 1 and hidden platform in the target quadrant on days 2–6 as a learning trial. i) Representative swimming traces during the probe trial session. j) Time spent in the target quadrant and k) target number of crossings where the hidden platform was previously placed during probe trial. All data are represented as mean ± SEM. *N* = 8–12 each. Statistical significance was determined via a one‐way ANOVA with a e,f,g,k) Tukey post hoc test and a two‐way ANOVA with a h) Tukey post hoc test. *p < 0.05 and ***p < 0.001 versus sham/control group; #*p* < 0.05 and ##*p* < 0.01 versus stroke/control group.

Using a multi‐LED array, we extended the brain PBM approach to evaluate the improvement of the cognitive function in the chronic phase poststroke. The mice were subjected to middle cerebral artery occlusion (MCAO)/reperfusion for 45 min to produce consistent selective lesions in the cortex, striatum, and hippocampus, resulting in low mortality, as described in **Figure** **5**a,b. Because the hippocampus largely contributes to long‐term memory deficit after stroke, the efficacy of brain PBM is clearly accessible. As shown in Figure 5c, the multi‐LED array (630 nm) was implanted and periodic PBM therapy was performed daily for 7 days, and all behavioral tests were performed until 28 days after ischemic brain injury.

In this experiment, the elevated plus maze was used to study anxiety levels, as shown in Figure 5d and Figure S6b (Supporting Information). Mice generally prefer to stay in the closed arms, and they demonstrate fear of the height of the maze. However, the stroke/control mice expressed a significantly reduced anxiety‐phenotype, which was characterized by the observation that they were spending more time in open arms, compared to the sham/control groups (1.56 ± 0.69% vs 4.92 ± 1% min in the sham/control and stroke/control groups, respectively, *p* < 0.05 [*p* = 0.023]; Figure 5e); this phenotype was not reversed by PBM treatment. In addition, no other significant differences in the total traveled distance were observed among all groups as shown in Figure 5f, suggesting that the brain PBM does not affect the mice's mobility. We further demonstrated whether brain PBM using a 630‐nm multi‐LED array could improve spatial learning and memory deficits in the stroke model using the Morris water maze test (Figure 5g). In the trial for testing spatial learning ability, the escape latency was defined as the time required for the mice to swim to a submerged platform (Figure S6c, Supporting Information). As shown in Figure 5h, stroke/control mice took longer to find the platform compared to the sham/control groups (*p* < 0.001), while the PBM‐treated stroke mice demonstrated an apparently shorter latency to find the hidden platform (*p* < 0.01 [*p* = 0.008]). In the probe trial test on the following day (day 7), the stroke/control mice spent less time in the target zone (*p* < 0.05 [*p* = 0.023]; Figure 5i,j) and crossed to the target zone significantly fewer times (*p* < 0.05 [*p* = 0.026]; Figure 5k) after the removal of the platform. PBM‐treated stroke mice showed a significantly increased time spent in the target zone (*p* < 0.05 [*p* = 0.016]) and crossed to the target significantly more frequently (*p* < 0.05 [*p* = 0.011]) than stroke/control mice (Figure 5i–k). These results indicate that brain PBM (i.e., 630‐nm wavelength) performed in the acute phase of stroke improved the spatial learning and memory, but did not recover the anxiety behavior in the chronic phase of stroke.

### 2.6 Effects of Brain PBM on AIM2 Inflammasome Activation and Pyroptosis in Stroke Mice

**Figure 6:**
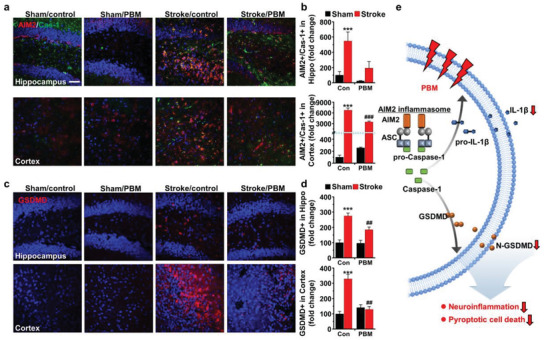
Effect of 630‐nm brain PBM using a multi‐LED array on AIM2 inflammasome and pyroptosis in the hippocampus and cortex following ischemic brain injury. a,b) Representative photographs and quantification graphs of AIM2^+^/Cas‐1^+^ and c,d) GSDMD (pyroptosis marker). AIM2 and GSDMD were labeled with red fluorescence, Cas‐1 was labeled with green fluorescence and DAPI was labeled with blue fluorescence. Quantification of AIM2^+^/Cas‐1^+^ and GSDMD fluorescence integrated optical density in the hippocampus and cortex. Scale bar = 50 µm. All data are represented as mean ± SEM. *N* = 3 each. Statistical significance was determined using a one‐way ANOVA with a Tukey post hoc test. ***p < 0.001 versus sham/control group; ##*p* < 0.01 and ###*p* < 0.001 versus stroke/control group. e) Schematic illustration of the effect of 630 nm brain PBM on AIM2 inflammasome signaling pathway. Brain PBM may prevent the neuroinflammation and pyroptotic cell death via inhibiting the activation of AIM2 inflammasome and related signaling pathway.

Focusing on the specific wavelength of 630 nm, we investigated the beneficial effect of brain PBM based on the expression of AIM2, caspase‐1, and gasdermin D (GSDMD), a pyroptosis marker, in the hippocampus and cortex. Compared to the sham/control group, a higher intensity of AIM2^+^/caspase‐1^+^ staining in the hippocampus and cortex was observed in the stroke/control group (7 days post stroke). Notably, as displayed in **Figure** **6**a,b, consistent with the results of the cognitive function tests, PBM‐treated stroke mice showed an apparent reduction of AIM2^+^/caspase‐1^+^ expression in the hippocampus and cortex (hippocampus: 452.82 ± 85.75% vs 634.41 ± 118.56%, stroke/PBM and stroke/control group, respectively; cortex: 938.96 ± 209.33% vs 4295.28 ± 634.35%, stroke/PBM and stroke/control group, respectively, *p* < 0.001). In addition, similar results were observed with GSDMD staining. Stroke/control mice showed robust staining for GSDMD in both the hippocampus and cortex, which was significantly reduced in the PBM‐treated group at 7 days post stroke compared to the stroke/control group (hippocampus: 184.81 ± 16.95% versus 275.12 ± 19.25%, stroke/PBM and stroke/control group, respectively, *p* < 0.01 (*p* = 0.007); cortex: 129.04 ± 18.41% versus 328.40 ± 30.13%, stroke/PBM and stroke/control, respectively, *p* < 0.01 (*p* = 0.001)), as presented in Figure 6c,d. Overall, the implanted multi‐LED array equipped with a 630‐nm wavelength was obviously effective in the acute phase of stroke by attenuating AIM2 inflammasome activation and mediating pyroptosis at the injured site at 7 days post stroke, as schematically demonstrated in Figure 6e.

### 2.7 Brain PBM‐Derived Modulation of Microglia Polarization to an Anti‐Inflammatory Phenotype in Stroke Mice

**Figure 7:**
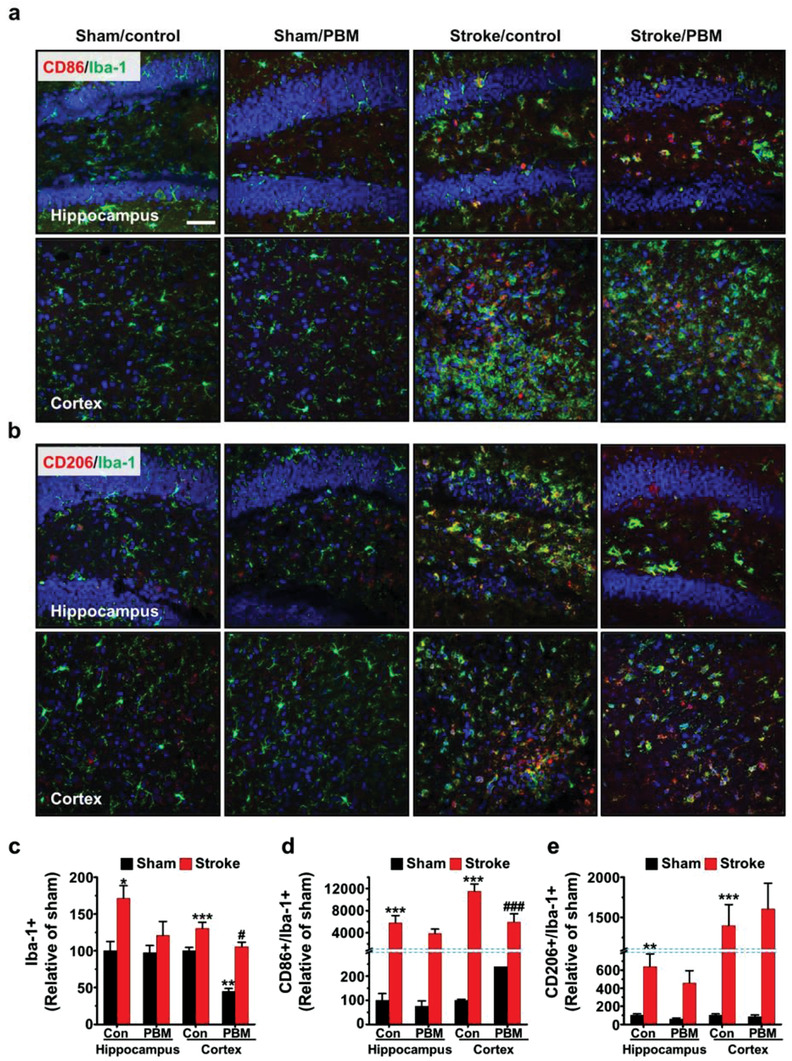
Effect of 630 nm brain PBM using multi‐LED array on microglia polarization in the hippocampus and cortex following ischemic brain injury. a) Double immunostaining of Iba‐1 (green) with CD86 (red) or b) CD206 (red) in the hippocampus and cortex. DAPI is labeled with blue fluorescence. Scale bar = 50 µm. c–e) Quantification of Iba‐1^+^, CD86^+^/Iba‐1^+^, and CD206^+^/Iba‐1^+^ fluorescence integrated optical density in the hippocampus and cortex. All data are represented as mean ± SEM. *N* = 3 each. Statistical significance was determined using a one‐way ANOVA with a Tukey post hoc test. **p < 0.01 and ***p < 0.001 versus sham/control group; #*p* < 0.05 and ###*p* < 0.001 versus stroke/control group.

**Figure 8:**
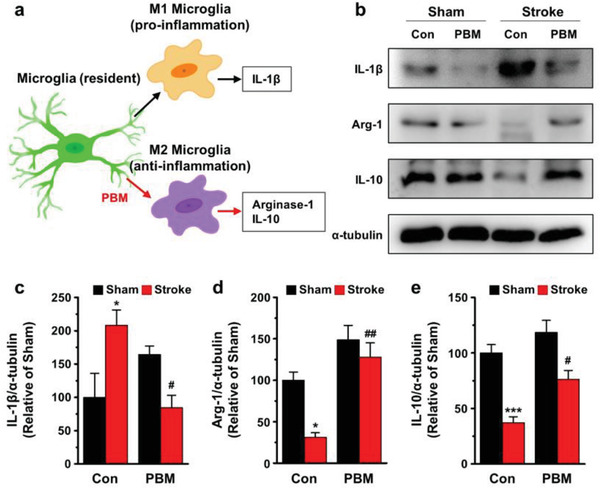
Effect of 630‐nm brain PBM using a multi‐LED array on proinflammatory and anti‐inflammatory cytokine production following ischemic brain injury. Protein levels of ischemic brains are examined using western blot analysis the next day after the last PBM treatment. a) Schematic illustration of the effect of 630‐nm brain PBM on microglia polarization. The resident microglia activate two types of polarization by stroke, and PBM treatment may inhibit the production of M1 phenotype microglia and reduce proinflammatory cytokine (IL‐1*β*). b) Representative western blot images of IL‐1*β* and anti‐inflammatory cytokines (Arg‐1 and IL‐10) in the ipsilateral lesion after stroke. c–e) Quantitative immunoblots of IL‐1*β*, Arg‐1, and IL‐10. Internal protein is normalized to *α*‐tubulin. All data are represented as mean ± SEM. *N* = 3–4 each. Statistical significance was determined via a one‐way ANOVA with a Tukey post hoc test. *p < 0.05 and ***p < 0.001 versus sham/control group; #*p* < 0.05 and ##*p* < 0.01 versus stroke/control group.

The next approach for the implantable brain PBM (i.e., 630 nm) was to examine microglia polarization in the ischemic mice. In this experiment, Iba‐1 (microglia marker) was double‐stained with CD86 (M1 surface marker) or CD206 (M2 surface marker) for the hippocampus and cortex. As presented, the visualized images substantiated clearly different intensity levels for the Iba‐1^+^, CD86+/Iba‐1^+^, and CD206^+^/Iba‐1^+^ cells in the stroke/control groups compared to the sham/control groups. The PBM treatment suppressed the expression of Iba‐1^+^ (105.41 ± 6.10% vs 130.41 ± 8.24%, stroke/PBM and stroke/control, respectively, *p* < 0.05 (*p* = 0.012); **Figure** **7**c). The double‐stained cells showed different trends in the intensity levels for CD86^+^/Iba‐1^+^ (5960.95 ± 1466.21% vs 11 520.42 ± 1279.07%, stroke/PBM and stroke/control, respectively, *p* < 0.001, Figure 7a,d). It was associated with stroke/control mice, but had no effect on cortical CD206+/Iba‐1+ (Figure 7b,e). The collective set of data suggested that reduced Iba‐1^+^ microglia by brain PBM was the result of decreasing M1 phenotype microglia, not the changing M2 phenotype microglia in the cortex. Therefore, we inferred possible serial processes in the inflammatory system, as schematically depicted in **Figure** **8**a. Finally, as shown in Figure 8b–e, the microglia polarization induced by brain PBM was confirmed by the increased production of IL‐1*β* (i.e., proinflammatory cytokine) and reduced arginase‐1 and IL‐10 (i.e., anti‐inflammatory cytokines), which were markedly reversed in the PBM‐treated group at 7 days post stroke. The conclusive results demonstrated that effective PBM at a wavelength of 630 nm in the acute phase of stroke appropriately modulated microglia polarization to produce anti‐inflammatory cytokines, which may facilitate long‐term cognitive recovery.

## 3 Discussion

In this study, we developed a lightweight and compact system of miniaturized electronic devices that incorporates flexible substrate and uses a simple packaged LED design to conduct in vivo experiments of brain PBM for a small animal. Using this device platform, we evaluated the beneficial effects of brain PBM on tissue and functional recovery following ischemic stroke. Among the various wavelength ranges, the wavelength of 630 nm was the most effective in reducing the direct infarction volume and neurological and motor impairment after ischemic brain injury. In addition, the implanted brain PBM with a 630‐nm LED array in the acute phase of stroke showed remarkably improved capability of spatial learning and memory in the chronic phase of stroke. Furthermore, PBM treatment with 630‐nm LEDs attenuated AIM2 inflammasome activation and inflammasome‐mediated pyroptosis and modulated microglial polarization in the hippocampus and cortex 7 days following ischemic stroke. Therefore, the ipsilesional cortical application of the classified PBM device at a certain wavelength (i.e., 630 nm) prevented tissue and functional damage in the acute ischemic brain and obviously attenuated the development of cognitive impairment in poststroke chronic phase through the combinatorial regulation of AIM2 inflammasome‐mediated pyroptosis and microglia polarization.

To date, extensive studies have suggested that brain PBM therapy may have positive effects on stroke lesions, and in most cases, the approaches have been focused on the scale of the wavelength, focused irradiation, energy density, and periodic duration.^[^ ^45^, ^46^ ^]^ As a more advanced and practical approach, detailed experimental parameters were chosen, and the relevant mechanisms were further elucidated by in vivo studies with LLLT using a multi‐LED stimulator as follows. First, to determine the suitable wavelength for the prevention or treatment of ischemic stroke, mice in the in vivo studies were exposed to three different wavelengths (630, 850, and 940 nm) on a fixed frequency (i.e., 20 min, twice a day for 3 days, before or after ischemic brain injury), as presented in Figure 3b,c. Notably, among the prepared sets of multi‐LEDs, PBM at a specific wavelength (i.e., 630 nm) represented the best preventive effect on the tissue and functional outcomes after ischemic brain damage (Figure 3c,d); furthermore, PBM with 630‐nm LLLT was the most therapeutically effective for functional recovery in stroke rehabilitation (Figure 3f,g). Recently, it has been reported that PBM with wavelengths in the ≈600 nm range has protective or therapeutic effects in rabbits, rats, and mice in stroke models.^[^ ^27^, ^28^, ^29^, ^30^, ^47^, ^48^ ^]^ However, pre‐ and post‐treatment of PBM with 940‐nm LLLT also showed functional improvement after ischemic stroke as presented in Figure 3, but the effect was weaker than that of 630‐nm PBM. On the other hand, the 850‐nm LLLT was not sufficiently effective in our model system although there have been several case studies with progressed results for stroke patients, based on 808‐nm PBM treatment, as reported previously.^[^ ^31^ ^]^ This is surprising by the fact that even slight wavelength differences have unexpected results. As the most studied wavelength range for stroke research is 808‐nm PBM, another evaluation using those wavelength ranges will be follow‐up investigations related to appropriate light sources (i.e., micro LED chips) and irradiation parameters. Indeed, it is worth noting that the optimized optical power input of PBM with 630‐nm‐based LLLT facilitated improvements in ischemic brain deficit on the supplied current of 20 mA in the operation of multi‐LED array for power density of ≈17 mW cm^−2^, indicating the most remarkable improvement in the tissue and functional outcomes. For comparison, it was also conducted with other inputs, such as 10 or 30 mA, in the in vivo PBM processes (data not shown). Therefore, we chose to apply a multi‐LED array at a 630‐nm wavelength (≈17 mW cm^−2^) for the subsequent PBM experimental schemes.^[^ ^48^ ^]^

As reported previously, the preservation of cerebrovascular blood vessels and reduction of neuroinflammation through glial cells are important recovery strategies after ischemic stroke.^[^ ^49^ ^]^ To this end, the brain PBM therapy increases the cerebral blood flow and metabolic capacity of the neurons and promotes neuronal protection and survival through anti‐inflammatory, anti‐apoptotic, and antioxidant responses.^[^ ^27^, ^28^, ^29^, ^30^, ^31^, ^32^ ^]^ Figure 4 shows that PBM with 630‐nm LLLT increased endothelial cells and decreased astrocytes and microglia in stroke‐damaged lesions. Our findings are consistent with a previous report on 610‐nm‐based PBM that showed protection of the blood‐brain barrier with reduced neuroinflammation by decreasing astrocytes and microglia, and reducing the expression of inflammatory mediators after ischemic brain injury.^[^ ^29^ ^]^ Overall, brain PBM using an implanted multi‐LED array with a wavelength of 630 nm alleviates tissue damage, as well as minimizes functional deficit by preserving blood vessels and attenuating glial cell activation after focal cerebral ischemia.

We recently reported that AIM2 inflammasome‐mediated inflammation and pyroptosis possibly contribute to PSCI.^[^ ^35^ ^]^ Based on the results, we hypothesized that the PBM approach could improve PSCI with a proven protective effect on acute brain injury at a 630‐nm wavelength. As expected, brain PBM in the acute phase of ischemic stroke induced beneficial effects on spatial learning and memory in the chronic phase of stroke, as presented in Figure 5. Moreover, 7 days after ischemic brain injury, activated‐AIM2 inflammasome and pyroptosis were inhibited by our PBM treatment (Figure 6a–d). To date, the pronounced effect of brain PBM on improving cognitive function has been reported in several studies. For example, the PBM approach based on a wavelength of 610 nm at an early stage of disease onset has been shown to improve learning and spatial memory by increasing amyloid‐*β* degradation in an Alzheimer's disease animal model (i.e., 5XFAD mice).^[^ ^50^ ^]^ In addition, reports suggest that PBM with 627 and 660 nm wavelengths improved spatial memory in an A*β*25‐35 peptide‐induced mouse model^[^ ^51^ ^]^ and in 18‐month‐old BALB/c mice,^[^ ^52^ ^]^ respectively. Similar to these results, our PBM with 630‐nm LED arrays, especially in the acute phase of stroke, attenuated AIM2 inflammasome activation and mediated pyroptosis in the injured regions, improving spatial learning and memory.

Postischemic inflammation is a hallmark of ischemic stroke pathology that plays a critical role in acute brain damage and has profound implications in long‐term recovery.^[^ ^53^ ^]^ The inflammatory response is regulated by M1 and M2 polarization of microglia, and the failure of the M1/M2 phenotype balance in inflammation‐associated injury can lead to chronic inflammation and secondary damage.^[^ ^54^ ^]^ As presented in Figure 7, we observed that our PBM treatment with a 630‐nm wavelength clearly reduced microglia (Iba‐1^+^) in the ischemic cortex. A detailed observation of microglia suppressed by PBM revealed reduced M1‐phenotype microglia (CD86^+^/Iba‐1^+^) without altering M2‐phenotype microglia (CD206^+^/Iba‐1^+^). Moreover, the PBM approach inhibited proinflammatory cytokine IL‐1*β* and increased the anti‐inflammatory cytokines, Arg‐1 and IL‐10 (Figure 8). Similar to these results, previous studies showed that PBM treatment at 610‐nm wavelength reduced IL‐1*β* and IL‐18 levels at 72 h post stroke; we also recently reported that AIM2 activation and other inflammasome signaling, including IL‐1*β*, could be involved in the development of PSCI during the subacute phase post stroke.^[^ ^27^, ^36^ ^]^ Therefore, the collective set of results indicates that inhibiting AIM2 inflammasome‐mediated proinflammatory response and promoting anti‐inflammatory response using brain PBM with a wavelength of 630 nm may contribute to recovering cognitive impairment after ischemic stroke.

## 4 Conclusion

In conclusion, ipsilesional cortical application of brain PBM using an implantable multi‐LED array may prevent tissue and functional damage in acute ischemic stroke injury and attenuate the development of cognitive impairment after stroke through regulation of AIM2 inflammasome‐mediated pyroptosis and microglia polarization. Therefore, brain PBM therapy has the potential to develop into a safe and effective neuroprotective treatment for patients with acute stroke damage and PSCI. Based on this basic research, clinic or home‐based PBM therapy using scalable manufacturing strategies for LED devices could be a promising strategy for neurorehabilitation in the future.

## 5 Experimental Section

### Multi‐LED Array Preparation and Characterization

The flexible substrate used in this study was PI that has sputtered Cu layers on both sides. Primary and secondary metallization was performed via holes. The initial size of the PI substrate was 10 × 10 cm, and the thickness of the PI and Cu patterns were 25 and 1 µm, respectively. A conventional photolithography/etching process was used to pattern the Cu layers. Next, packaged LED chips (Wurth Electronics, Niedernhall, Germany) were manually placed on the contact pads after applying solder paste (i.e., Sn3.0Ag0.5Cu [Kester, Itasca, IL, USA]). The reflow process was set at a temperature of ≈250 °C with a melt interval duration of ≈1 min. Then, the thin layers of an overlay (12 µm, PI with epoxy adhesive) were conformed and bonded to package the top/bottom planes, protecting the exposed surface of the electrodes on the circuit, except for the LED components. Finally, the individual multi‐LED chips were separated into a desired design using an ultraviolet laser cutting machine. A water‐proof PDMS encapsulant was then applied using a dip‐coating process on the surface‐mount LEDs (SYLGARD 184; Dow, Midland, MI, USA; 10:1 mixing ratio of prepolymer and curing agent), which were cured in an oven at 80 °C for 1 h. This encapsulation can protect the solder joints, resulting in a reliable evaluation before implantation. The performance of the LEDs and interconnected stabilities were measured by a semiconductor parameter analyzer (Keithley 4200A; Keithley, Beaverton, OR, USA), and the mechanical durability of the multi‐LED array was repeatably tested by loading pressure in a folded state (CT3 analyzer; Brookfield, Middleboro, MA, USA).

### Multi‐LED Array Implantation

For the epicranial implant, the scalp and underlying tissues were removed, and the multi‐LED array was implanted using general dental cement (DurelonTM; 3M ESPE, Seefeld, Germany). The multi‐LED array was positioned over the sensorimotor cortex (Figure 1a–c; Figure S7, Supporting Information) of the ipsilesional region.

### Animal Experiments and Experimental Procedures

Male C57BL/6 mice were purchased from Hana Biotech (Ansan, Korea). Mice were housed under a 12‐h light/dark cycle and allowed ad libitum access to food and water. The animal protocol used in this study was reviewed and approved by the Pusan National University – Institutional Animal Care and Use Committee (PNU‐IACUC) as per their ethical procedures and scientific care (PNU‐2019‐2182, PNU‐2019‐2390). For the effect of brain PBM on acute brain injury, 630, 850, or 940 nm PBM was repeated twice a day before or after focal cerebral ischemia for 3 days over the ipsilateral cortical region (Figure 3b,e). Two types of brain PBM were used depending on whether it was for prevention or treatment. One type of PBM was performed twice a day from 3 days before ischemic brain injury to determine the preventive effect (Figure 3b) and the other type was performed immediately after focal cerebral ischemia to assess the therapeutic effect of PBM (Figure 3e). For evaluating the effect of PBM on PSCI, brain PBM was performed once daily for 7 days over the ipsilateral cortical region (Figure 5c). PBM was performed using a 630‐nm LED array, which emits ≈17 mW cm^−2^ light. PBM current was applied continuously for 20 min using a bench power supply (PST‐3202; GW INSTEK, New Taipei City, Taiwan) under anesthesia. The same set of experimental conditions were provided to minimize the comparability issues with repetitive anesthesia, in the efficacy study of PBM. While the stimulated groups were anesthetized, the control groups were also equally anesthetized for 20 min without LED irradiation. Anesthesia was achieved by a nose cone‐delivered isoflurane (1.5% maintenance in 80% N_2_O and 20% O_2_). Rectal temperature was maintained at 36.5–35.5°C using a Panlab thermostatically controlled heating mat (Harvard Apparatus, Holliston, MA, USA).

### Photothrombotic Stroke Model

Photothrombotic cortical ischemia was induced by photothrombosis of the cortical microvessels, as previously described (Figure 3a; Figure S6, Supporting Information).^[^ ^54^ ^]^ Briefly, mice were anesthetized with 2% isoflurane in 20% O_2_ and 80% N_2_O; subsequently, they received an intraperitoneal (i.p.) injection of Rose Bengal (Sigma‐Aldrich, St. Louis, MO, USA; 0.1 mL of 10 mg mL^−1^ in 0.9% saline) 5 min prior to illumination. Each mouse was fixed on a stereotaxic frame (David Kopf Instruments, Tujunga, CA, USA), and its skull was exposed. A fiber‐optic bundle containing a CL6000 LED cold light source (Carl Zeiss, Jena, Germany) was positioned onto the sensorimotor cortex of the exposed skull (2.4 mm lateral from the bregma) and illuminated for 15 min (Figure S8, Supporting Information). The scalp was sutured after illumination. The infarct volume, neurological score, and motor function test were performed according to the defined experimental schedule (Figure 3b,e).

### Middle Cerebral Artery Occlusion/Reperfusion Stroke Model

For the PSCI model, the transient focal cerebral ischemia was induced using MCAO/reperfusion as per the previously described intraluminal filament technique and maintained for 28 days (Figure 5a–c).^[^ ^35^ ^]^ Briefly, the mice were anesthetized with isoflurane (2% for induction and 1.5% for maintenance, in 80% N_2_O and 20% O_2_) through respiratory anesthesia via face mask. A fiber‐optic probe was affixed to the skull above the left middle carotid artery (MCA) to continuously monitor the regional cerebral blood flow during the surgery and at reperfusion using the PeriFlux Laser Doppler System 5000 (Perimed, Stockholm, Sweden). In the MCAO surgical procedure, a silicon‐coated 7‐0 monofilament (Duccol Corporation, Redlands, CA, USA) was placed in the internal carotid artery, and then the monofilament occluded the MCA to induce MCAO. After 45 min, the monofilament was withdrawn to allow reperfusion and monitored using the laser Doppler flowmeter (Perimed, Stockholm, Sweden).

### Infarct Volume

The infarct size was determined via 2, 3, 5‐ TTC staining of 2‐mm‐thick brain sections. Infarct size was quantified using i‐Solution software (Image & Microscope Technology, Vancouver, Canada). Measurements of the direct infarct volume included areas of the ipsilateral side that had sustained direct damage.

### Neurological Score

Neurological deficits were evaluated using the following scoring system: 1 = turning in the direction of the ipsilateral (non‐damaged) side when held by the tail; 2 = turning in the direction of the contralateral (damaged) side and difficulty bearing weight; 3 = unable to bear weight on the contralateral side; 4 = no spontaneous movement.^[^ ^55^ ^]^

### Wire‐Grip Test

Vestibular motor function was evaluated using the wire‐grip test (Figure S9, Supporting Information). Each mouse was suspended on a metal wire and forced to hang using both forepaws. Wire grip was scored as follows: 1 = not holding onto the wire; 2 = holding onto the wire using both forepaws and hind paws but not the tail; 3 = holding onto the wire using both forepaws and hind paws as well as the tail, without movement; 4 = moving on the wire using both forepaws, both hind paws, and tail; 5 = moving well on the wire.^[^ ^55^ ^]^

### Elevated Plus Maze Test

The elevated plus maze was tested to measure anxiety‐like behavior (Figure S6b, Supporting Information).^[^ ^56^ ^]^ The test was conducted by placing the mouse on a central zone of a maze consisting of two open arms (without walls, 27‐cm long, 5‐cm wide, and elevated 35 cm above the floor) and two closed arms (with walls). The mouse movements were recorded for 10 min and analyzed using a video tracking system (Panlab, Barcelona, Spain).

### Morris Water Maze Test

The Morris water maze was used to measure spatial learning and memory (Figure S6c, Supporting Information).^[^ ^57^ ^]^ The mouse explored the maze to find the circular (10 × 10 cm) target platform in a pool (120 cm diameter, 50 cm deep). The cues to help the mouse find the target platform were attached to the pool wall above the water surface near the target platform. The test was conducted for 7 consecutive days. On day 1, all mice were allowed to explore in four trials (90 s per trial), to recognize cues and the visible platform. Each trial had a different starting point within the pool, but the location of the platform and cues was fixed. If the mouse found the platform within 90 s, it stayed on the platform for 15 s. If the mouse did not find the platform within the time required, it was guided to the platform and stayed there for 30 s. On days 2–6, the mouse was placed in an opaque water‐filled pool (hidden platform) for learning trial tests, and the same procedure used on day 1 was followed. On day 7, the mouse was allowed to swim freely without the platform for 90 s for the probe trial test. The data were recorded using the Smart software (Panlab) and the percentage of time spent in each quadrant and the speed of movement was analyzed.

### Immunofluorescence Staining

Mice were perfused with cold PBS followed by 4% paraformaldehyde after behavioral assessment or PBM treatment. Immediately thereafter, the brains were harvested and further fixed for 24 h in 4% paraformaldehyde; subsequently, they were cryoprotected in 30% sucrose for 72 h at 4 °C. Each brain was frozen in optimal cutting temperature compound (Sakura Finetek, Torrance, CA, USA) and stored at −80 °C until analysis. The frozen brains were cut into sections 20 µm‐thick (for frozen sections) or 40 µm‐thick (for free‐floating sections) using a CM 3050 cryostat (Leica Microsystems, Wetzlar, Germany). The brain sections were immunostained with anti‐NeuN (1:100; Millipore, Burlington, MA), anti‐CD‐31 (1:100; BD Bioscience, Franklin Lakes, NJ, USA), anti‐GFAP (1:100, Dako, Santa Clara, CA, USA), anti‐Iba‐1 (1:100; Wako, Osaka, Japan), anti‐AIM2 (1:200; Invitrogen, Carlsbad, CA, USA), anti‐caspase‐1 (1:100; Novus, Centennial, CO, USA), anti‐gasdermin D (GSDMD, 1:100; Abcam, Cambridge, UK), anti‐CD86 (1:100; Abcam), and abti‐CD206 (1:100; Abcam) antibodies overnight at 4°C. Brain sections were subsequently incubated with Alexa 488 or Alexa 594‐conjugated secondary antibodies (1:500; Life Technologies, Carlsbad, CA, USA) for 2 h in total darkness. 4′,6‐Diamidino‐2‐phenylindole (DAPI; Molecular Probes, Eugene, OR, USA) was used for the staining of the nuclei. Fluorescence images were visualized using an FV1000 laser scanning confocal device (Olympus, Tokyo, Japan). The images were quantified using i‐Solution software (Image & Microscope Technology), and Image J software (NIH, Bethesda, MD, USA).

### Western Blot

The total protein was extracted from the brain following a standard procedure and then lysed using radioimmunoprecipitation assay (RIPA) buffer (Cell Signaling, Beverly, MA, USA) with a protease inhibitors mixture (Genedepot, Katy, TX, USA) and phosphatase inhibitors mixture (Genedepot). Thirty µg of protein was separated using 15% sodium dodecyl sulfate‐polyacrylamide gel electrophoresis (SDS‐PAGE) and then transferred onto a poly‐vinylidene‐difluoride (PVDF) membrane (Amersham, Little Chalfort, UK). The immunoblot analysis was performed with primary antibodies; anti‐IL‐1*β* (1:1000; Santa Cruz, Dallas, TX, USA), anti‐arginase‐1 (1:1000; Abcam), and anti‐IL‐10 (1:1000; Abcam). Then, immunoblots were incubated with secondary antibodies (1:4000; Enzo Life Science, Farmingdale, NY, USA) conjugated with horseradish peroxidase. *α*‐tubulin (1:10 000; Sigma‐Aldrich, St Louis, MO, USA) was used as a reference protein for quantitative analysis. The chemiluminescence intensity was captured using an ImageQuant LAS 4000 apparatus (GE Healthcare Life Sciences, Uppsala, Sweden) and measured using the ImageJ software (NIH).

### Statistical Analyses

The following steps were conducted for data pre‐processing. Before beginning experimental steps, validation and quality control process had been conducted for capturing raw data. Then, Shapiro‐Wilk test was conducted for verifying normality assumption of continuous data. Next, the possible outliers of result data based on Cook's distance for deciding additional sensitivity analyses were checked. All data were established through above pre‐processing process and then conducted statistical steps. Data are expressed as means ± standard error of the mean (SEM) and all statistical analyses were performed using SigmaPlot 11.2 (Systat Software Inc, San Jose, CA, USA). Two groups were compared using unpaired, two‐tailed Student“s *t*‐tests to determine the effect of 630 nm PBM on each brain cells following ischemic brain injury, whereas one‐ and two‐way analyses of variance (ANOVA) with Tukey's post hoc test was performed to test the effect of various wavelengths of PBM on ischemic stroke, and the effect of 630 nm PBM on cognitive dysfunction and AIM2 inflammasome‐mediated neuroinflammation following ischemic brain injury. A *p* < 0.05 was considered statistically significant. 

## Conflict of Interest

The authors declare no conflict of interest.

## Supporting information

Supporting Information

## Data Availability

The data that support the findings of this study are available in the supplementary material of this article.
