# Supplementary material for: Benefits of a Skull‐Interfaced Flexible and Implantable Multilight Emitting Diode Array for Photobiomodulation in Ischemic Stroke
Source: Adv Sci (Weinh). 2022 Jan 25;9(11):2104629. doi: 10.1002/advs.202104629 (PMC9008794; doi:10.1002/advs.202104629)
Supplement: Supplementary file 1 — Supporting Information [file ADVS-9-2104629-s001.pdf]

WILEY-VCH

ADVANCED SCIENCE
Open Access

# Supporting Information

for *Adv. Sci.,* DOI: 10.1002/advs.202104629

# Benefits of a Skull-Interfaced Flexible and Implantable Multi-Light Emitting Diode Array for Photobiomodulation in Ischemic Stroke

*Hyunha Kim, Min Jae Kim, Young Woo Kwon, Sangheon Jeon, Seo-Yeon Lee, Chang-Seok Kim, Byung Tae Choi, Yong-Il Shin, Suck Won Hong,* Hwa Kyoung Shin**

# Supporting Information

## Benefits of a Skull-Interfaced Flexible and Implantable Multi-Light Emitting Diode Array for Photobiomodulation in Ischemic Stroke

*Hyunha Kim, Min Jae Kim, Young Woo Kwon, Sangheon Jeon, Seo-Yeon Lee, Chang-Seok Kim, Byung Tae Choi, Yong-Il Shin, Suck Won Hong,* Hwa Kyoung Shin**

H. Kim, M. J. Kim, B. T. Choi, Prof. H. K. Shin
Department of Korean Medical Science
Graduate Training Program of Korean Medical Therapeutics for Healthy-Aging
School of Korean Medicine
Pusan National University
Yangsan, 50612, Republic of Korea.
E-mail: julie@pusan.ac.kr (H.K.S.)

H. Kim
Department of Pharmacology and Neuroscience
Creighton University School of Medicine
Omaha, NE 68178, USA

Y. W. Kwon
Department of Nano-Fusion Technology
College of Nanoscience & Nanotechnology
Pusan National University
Busan 46241, Republic of Korea

S. Jeon, Prof. C.-S. Kim, Prof. S. W. Hong
Department of Congo-Mechatronics Engineering
Department of Optics and Mechatronics Engineering
College of Nanoscience & Nanotechnology
Pusan National University
Busan 46241, Republic of Korea
E-mail: swhong@pusan.ac.kr (S.W.H.)

S.-Y. Lee
Department of Pharmacology
Wonkwang University School of Medicine
Iksan, 54538, Republic of Korea

Y.-I. Shin
Department of Rehabilitation Medicine
School of Medicine
Pusan National University
Yangsan, 50612, Republic of Korea

Keywords: photobiomodulation, stroke, light-emitting diode, post-stroke cognitive impairment

**Table S1.** Power density according to current applied to a flexible multi-LED array.

| **Power density (mW cm$^{-2}$)** | |
|---|---|
| Current (mA) | 630 nm |
| 15 | 12.44 |
| 20 | 17.04 |
| 25 | 20.64 |
| 30 | 24.92 |

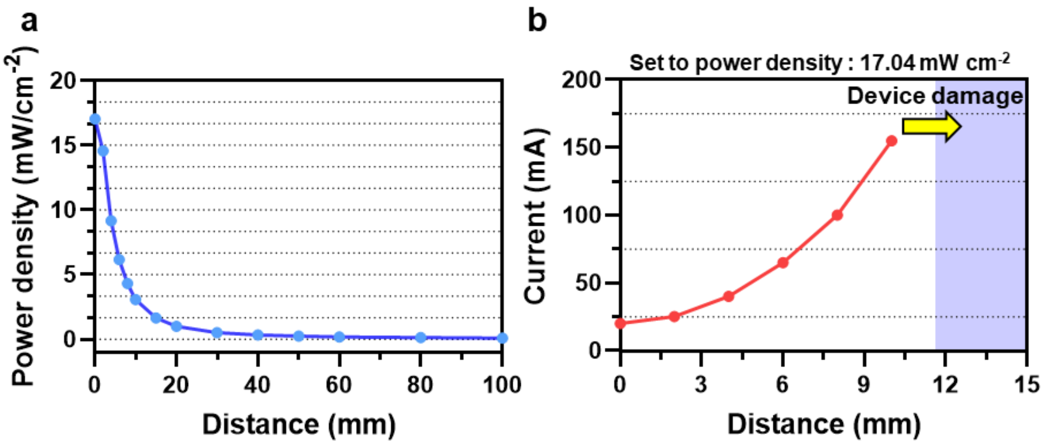


**Figure S1.** (a) Power density levels as a function of the distance between multi-LED arrays and the target region. (b) Operating current as a function of distance to maintain a constant power density (i.e., 630-nm LED, 17.04 mW $cm^{-2}$). Above the distance of ~12 mm from the target area, the permissible current was exceeded, causing permanent damage to the LEDs; the measured maximum surface temperature was found to be ~100 °C.

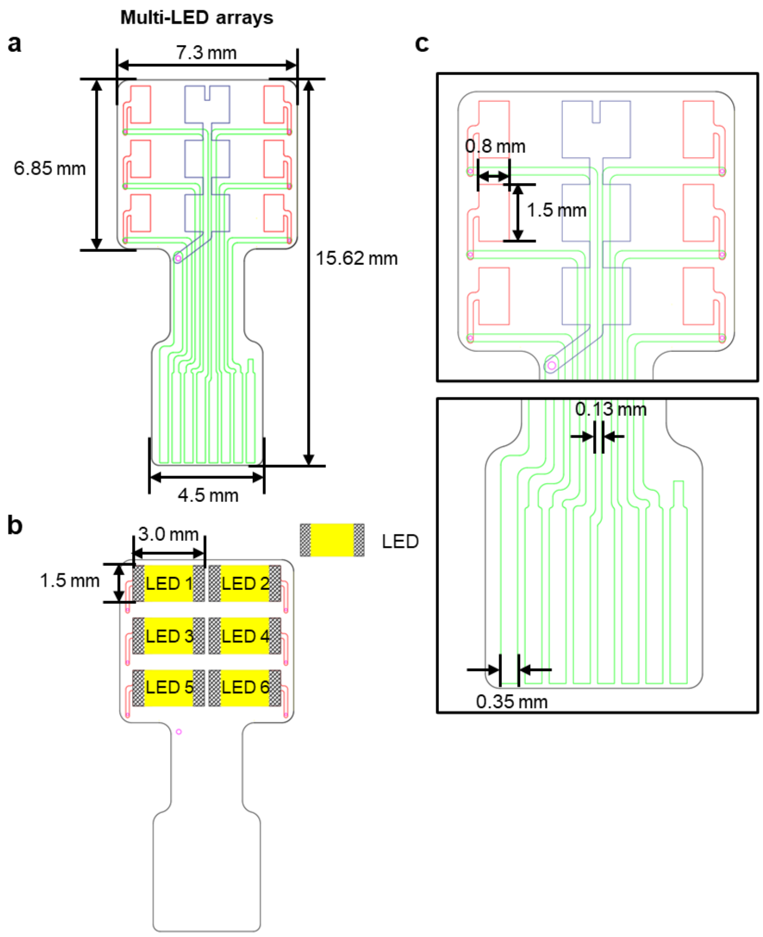


**Figure S2.** A circuit layout of the multi-LED array. (a) Actual dimensions of head and connection leads of a chip. (b) The size of the individual LEDs mounted on the front side. (c) Top: a magnified circuit layout of the front (red) and backplane (green). Bottom: the defined dimensions for plug-leads.

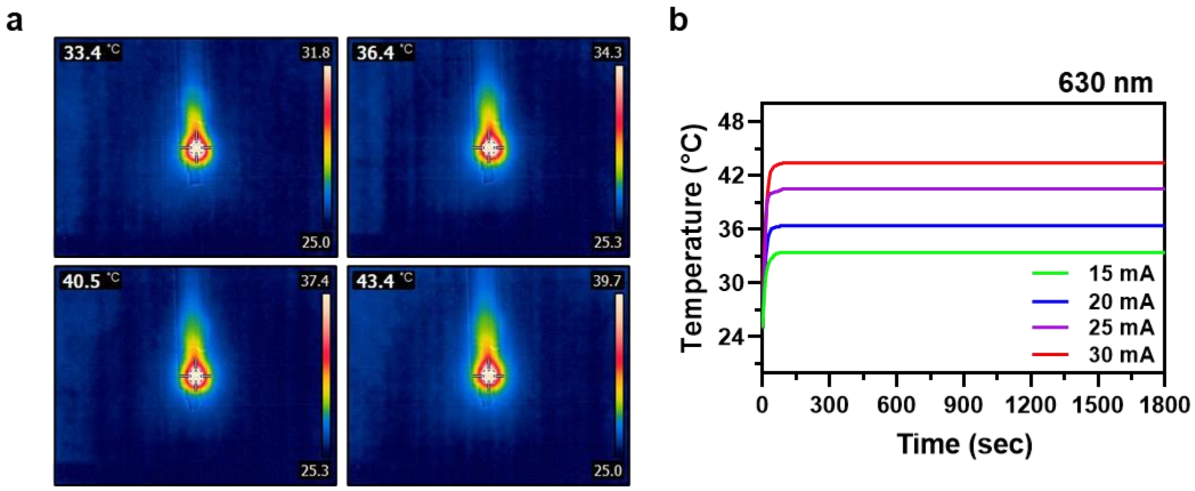


**Figure S3.** Measured surface temperature of multi-LED array during operation depending on the applied current ranges in air. Thermal IR images (a), and time-dependent temperature changes (b).

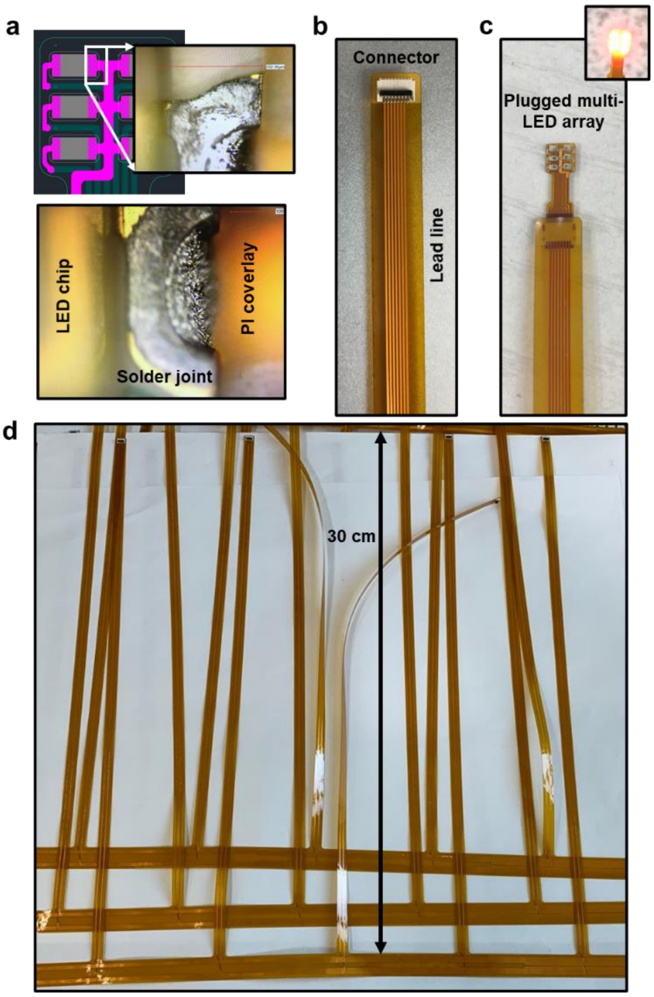


**Figure S4.** The main component for the brain PBM experiment using a multi-LED array. (a) Optical micrographs of the solder joint between the surface-mounted LED and PI top-plane coverlay, in which the gap was measured to be ~292 μm. (b-c) Digital images of the connector and plugged chip of the multi-LED array. (d) Digital image of the interconnection leads for supplying electrical power (the length of the leads is 30 cm).

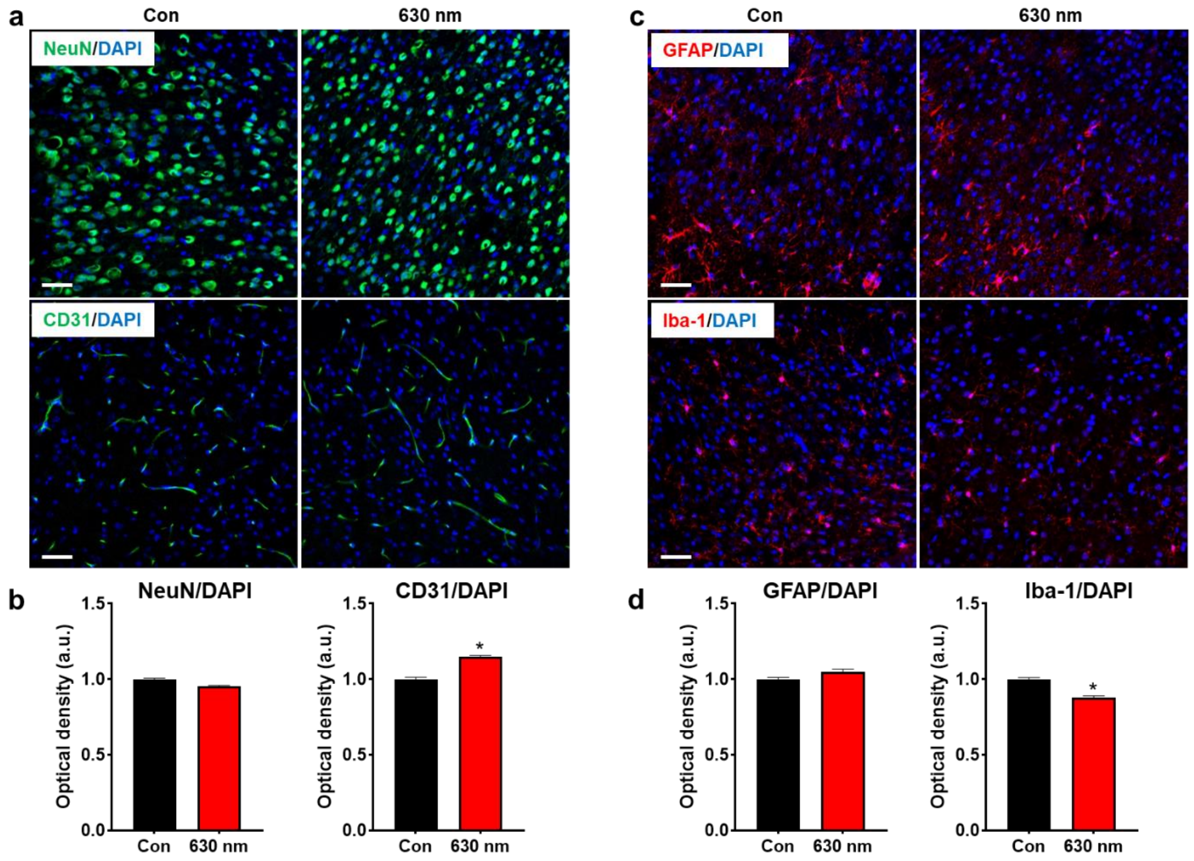


**Figure S5.** Effect of 630 nm brain PBM using multi-LED array on brain cells in the normal brain. Mice were treated with 630 nm PBM (~17 mW $cm^{-2}$, 20 min) twice a day for 3 days. (a) and (b) Representative photographs and quantification graphs of NeuN (green, neuronal marker) and CD31 (green, endothelial cell marker). (c,d) Representative photographs and quantification graphs of GFAP (red, astrocyte marker) and Iba-1 (red, microglia marker) at the cortical regions. DAPI is labeled with blue fluorescence. Scale bar = 50 μm. All data are represented as mean ± SEM. N = 3 each. Statistical significance was determined by unpaired, two-tailed Student‘s t-tests. *P < 0.05 vs. control group (Con).

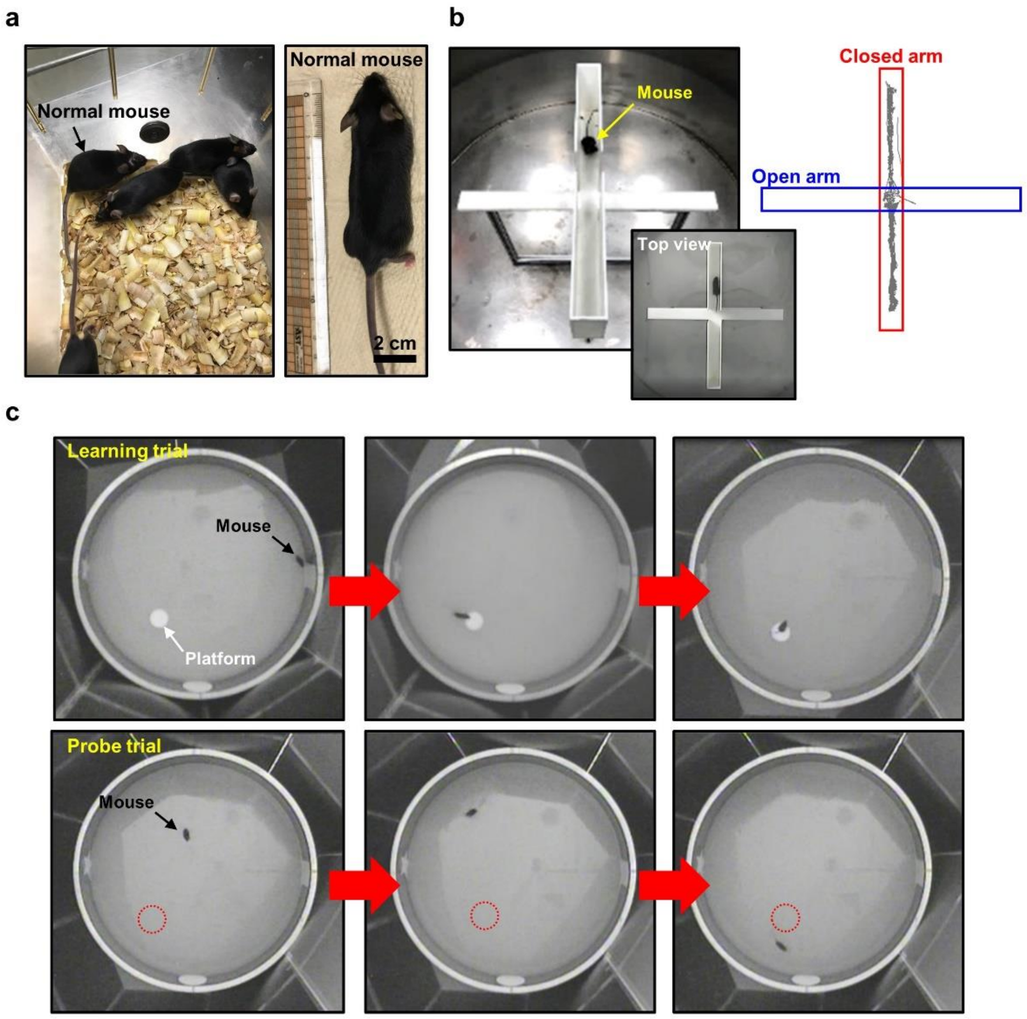


**Figure S6. (**a) Typical size of mouse used in the experiment. (b) Captured photographs of a moving mouse on the elevated plus-maze to study anxiety levels, and the related mapping position in tracking measurement from the top view (inset). (c) Captured photographs in learning and probe trial experiments.

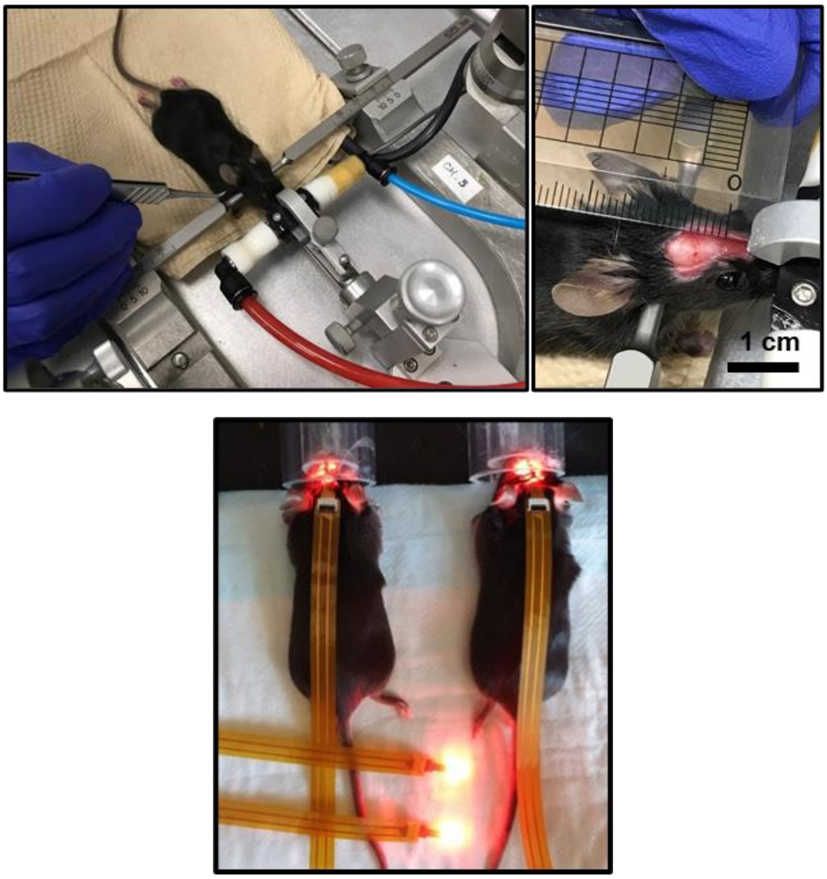


**Figure S7.** Photographs of an animal experiment using a stereotactic instrument (upper left), the surgical dimension for the implantation of the multi-LED array (upper right), and undergoing brain PBM using the implanted multi-LED array powered by the extended leads (bottom).

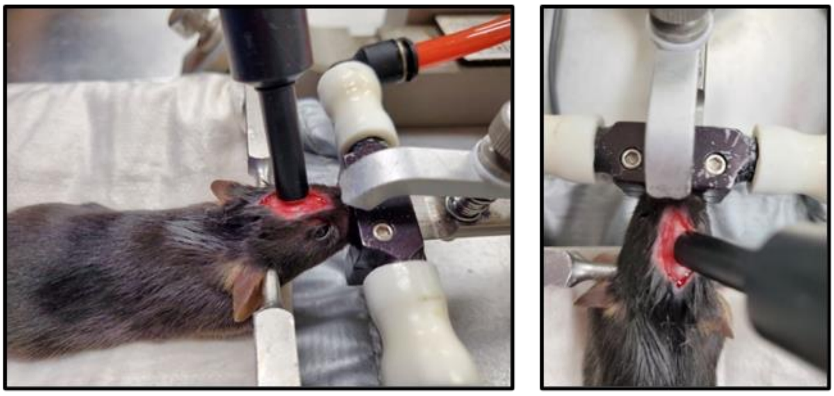

**Figure S8.** Photographs show the coagulation procedure of cortical microvessels by illuminating onto the sensorimotor cortex of exposed skull using a KL 1500 LED cold light source after tail injection of rose bengal.

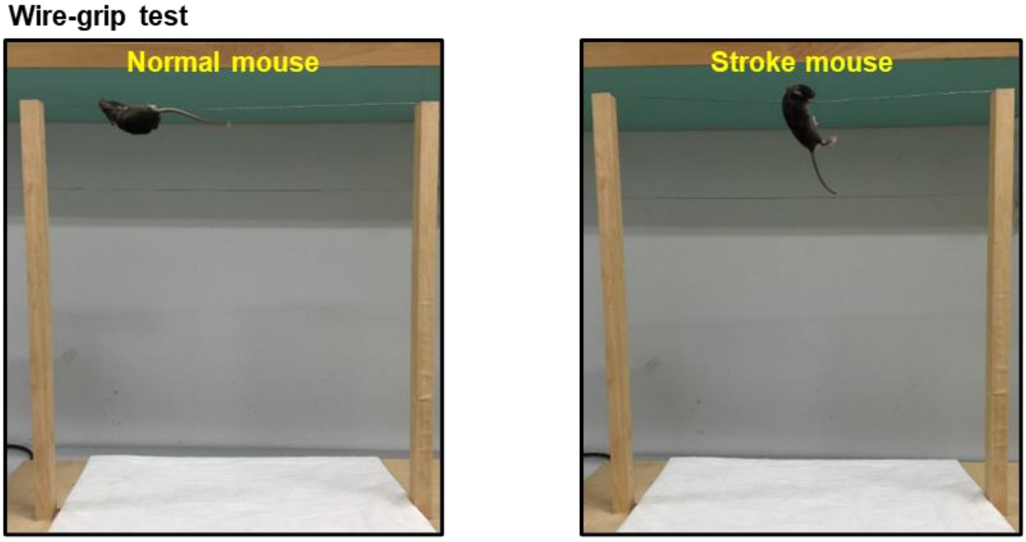


**Figure S9.** Photographs show the wire-grip test process for both normal and stroke mouse to evaluate the vestibular-motor function. Each mouse was suspended on a metal wire and forced to hang using both forepaws. Normal mouse uses the four limbs with tail, but stroke mouse is difficult using both forepaws and hind paws as well as the tail.
